# Increased native T1-values at the interventricular insertion regions of precapillary pulmonary hypertension patients

**DOI:** 10.1186/1532-429X-17-S1-Q44

**Published:** 2015-02-03

**Authors:** Onno A Spruijt, Harm-Jan Bogaard, Loek Vissers, Anton Vonk-Noordegraaf, Tim J Marcus

**Affiliations:** 1 Department of Pulmonary Medicine, VU University Medical Center, Amsterdam, Netherlands; 2 Department of Physics and Medical Technology, VU University Medical Center, Amsterdam, Netherlands

## Background

Due to pressure overload of the right ventricle (RV) in precapillary pulmonary hypertension (PH) patients, the interventricular insertion regions showed Late Gadolinium Enhancement (LGE), representing fibrosis (Blyth et al, Eur Heart J, 2005 Oct;26(19):1993-9). Another promising technique to characterize myocardium is native T1-mapping. Native T1-mapping can be assessed without contrast agents and the myocardial T1 can be quantified without the need of a reference area. Therefore, the aim of this study was to characterize the interventricular insertion regions in precapillary PH patients using native T1-mapping.

## Methods

70 precapillary PH patients (mean pulmonary artery pressure = 47±13mmHg) were included. Native T1-mapping was acquired on a Siemens 1.5 T Avanto scanner. A modified Look-Locker inversion-recovery (MOLLI) pulse sequence was used on a mid-ventricular short axis imaging plane. Three, three, and five non-segmented images were acquired at end-diastole of consecutive heart beats to sample the recovery of longitudinal magnetization after the inversion pulse. Minimal inversion time was 100 ms (Messroghli et al, JMRI 26:1081-1086, 2007). Motion compensation was applied. Native T1-values were assessed using regions of interest (ROIs) at the interventricular insertion regions, the RV free wall and left ventricular (LV) free wall.

## Results

**Figure 1:**
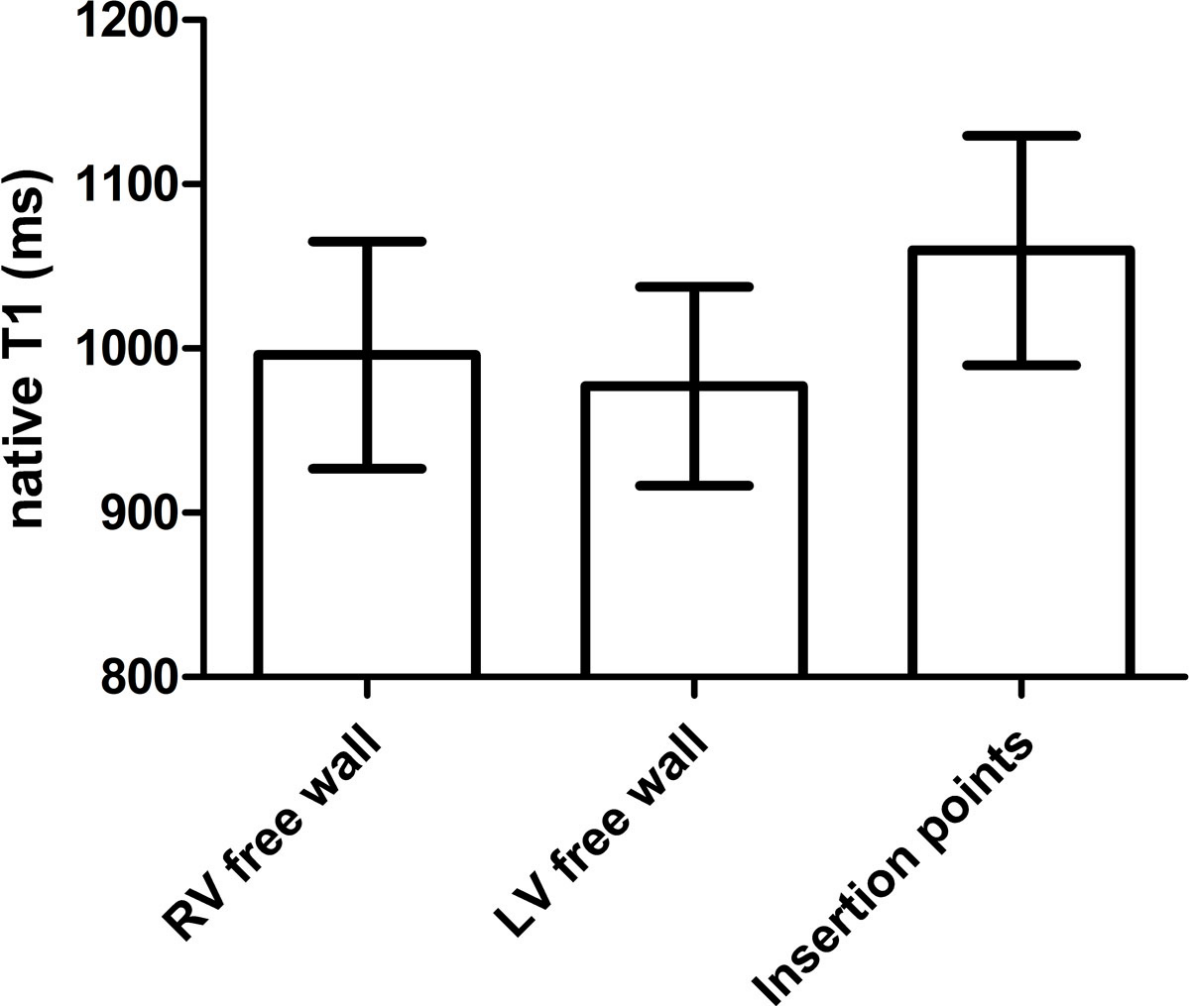
Data is presented as mean ± SD

Native T1-values of the RV were significantly higher than native T1-values of the LV (p=0,038). Native T1-values at the interventricular insertion regions were significantly higher compared to the RV free wall (p<0.001) and LV free wall (p<0.001) (Figure 1). Native T1-values at the insertion regions were significantly related to right atrial pressure (pearson r=0.310; p=0.01), RV end-diastolic volume (pearson r=0.376; p=0.001), RV ejection fraction (pearson r=-0.282; p=0.018) and NT pro-BNP (pearson r=0.392; p=0.001).

## Conclusions

Native T1-values at the interventricular insertion regions are significantly increased in precapillary PH and are related to disease severity. This finding is in line with previous PH studies using LGE where contrast-enhancement was observed in the same region. Our results show that native T1-mapping can be an alternative for the characterization of the interventricular insertion regions without the use of contrast agents.

## Funding

N/A.

